# Investigations of the pore formation in the lead selenide films using glacial acetic acid- and nitric acid-based electrolyte

**DOI:** 10.1186/1556-276X-7-338

**Published:** 2012-06-22

**Authors:** Sergey P Zimin, Egor S Gorlachev, Viktor V Naumov, Fedor O Skok

**Affiliations:** 1 Microelectronics Department, Yaroslavl State University, Yaroslavl, 150000, Russia; 2 Yaroslavl Branch of the Institute of Physics and Technology of Russian Academy of Sciences, Yaroslavl, 150007, Russia

**Keywords:** Porous semiconductors, Lead selenide, Anodic electrochemical treatment, Electrolyte, Mesopores, 81.05.Rm; 71.20.Nr; 81.65.Cf.

## Abstract

We report a novel synthesis of porous PbSe layers on Si substrates by anodic electrochemical treatment of PbSe/CaF_2_/Si(111) epitaxial structures in an electrolyte solution based on glacial acetic acid and nitric acid. Electron microscopy, X-ray diffractometry, and local chemical microanalysis investigation results for the porous layers are presented. Average size of the synthesized mesopores with approximately 10^10^ cm^−2^ surface density was determined to be 22 nm. The observed phenomenon of the active selenium redeposition on the mesopore walls during anodic treatment is discussed.

## Background

Currently, there is a new rapidly emerging direction of modern nanotechnology research in the fabrication of porous semiconductor compound materials. Such nanostructured materials can be applied in many novel and unique practical applications, such as optoelectronic devices based on the quantum size effects due to the small dimensions of the interpore material, or biomedical compounds based on storing nanoinclusions in the pore volume, and many more. Of course, porous Si [1] and III-V and II-VI compound semiconductors [2,3] to date have been studied rather extensively. On the other hand, IV-VI materials, in particular lead chalcogenides PbX (X = Te, Se, S), while being extremely useful for thermoelectric and optoelectronic applications [4,5], are not researched at porous form almost at all. Among the reasons for that are the lack of the large monocrystalline wafer samples fitted for anodic cell treatment and also the absence of an established electrolyte etchant solution. Recently, while being able to be the first to demonstrate the pore formation in lead chalcogenide layers and study their properties [6-9], we also found out that a potassium hydroxide (KOH)-based electrolyte, which was originally proposed by Norr [10,11] and is most commonly used for the PbX electropolishing, is not optimal for a stable pore formation in PbSe, in contrast to PbTe [9]. It became necessary to optimize anodic treatment conditions for the fabrication of a well-defined pore morphology in lead selenide layers, and such new and more fruitful approach is presented in this work.

## Methods

Initial samples were high-quality epitaxial monocrystalline PbSe films with a thickness of 2.2 to 5.2 μm grown on CaF_2_/Si(111) wafer substrates by molecular beam epitaxy (MBE) in ETH, Zürich [5]. The thickness of the calcium fluoride buffer layer was 2 to 4 nm. Anodic electrochemical treatment experiments were based on a technological approach that allows us to prepare porous lead chalcogenide layers on silicon wafer substrates using a vertical-type electrochemical cell [6]. The key treatment feature was the electrolyte solution. For the discussed study, the first batch of the initial epitaxial PbSe films was anodized using a KOH-based electrolyte solution (Norr electrolyte) [10] containing 20 g of KOH, 45 ml of distilled water, 35 ml of glycerol, and 20 ml of ethanol. Current density was 2 to 8 mA·cm^−2^, and treatment duration was 10 to 20 min, performed at room temperature. These film samples were further divided into two groups depending on the morphology type of their initial surface: the first group had a typical flat surface for MBE-grown PbX layers on CaF_2_/Si(111) with nanoterraces and dislocation exit pits with approximately 10^7^ cm^−2^ density, while the second group had a peculiar granular surface (for details, see [8]). The second batch of PbSe films (containing 3% Sn, with flat initial surface) was anodized using a solution of 10 ml of nitric acid (HNO_3_), 10 ml of glacial (undiluted) acetic acid (CH_3_COOH), and 40 ml of glycerol (first used by EH Tompkins and GL Johnson [11] for the electropolishing of lead selenide at high current densities), for which, by analogy with porous silicon, we reduced the current density (1 mA·cm^−2^) and processing temperature (20°C) with a duration of 10 min. Scanning electron microscopy (SEM) studies were performed on Supra-40 (Carl Zeiss, Inc., Oberkochen, Germany); chemical energy-dispersive X-ray spectroscopy (EDS) local microanalysis was carried out simultaneously with SEM using an INCA-Energy spectrometer (Oxford Instruments, Abingdon, UK).

## Results and discussion

**Figure 1:**
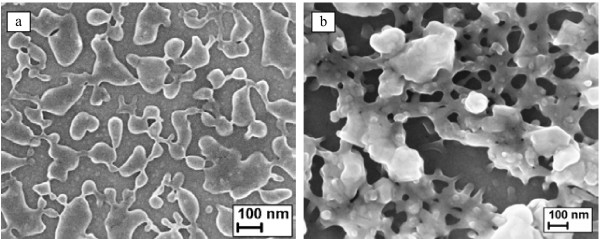
**SEM images of the modified PbSe layers obtained by anodic treatment with Norr electrolyte.** ( **a**) Island-like morphology for the samples with initial flat film surface. ( **b**) Hierarchical porous film for the samples with initial granular film surface.

The first batch of porous PbSe films was fabricated using the Norr electrolyte. We discussed these results in much detail in [8], and here, we will give only the basic overview. The principal result was that the modified anodized layer morphology strongly depended on the initial film surface state. Thus, PbSe layers with island-like morphology (Figure 1a) for the films with flat initial surface were obtained. PbSe nanoislands with a lateral size of 40 to 400 nm, a height of 90 nm, and average separating gaps of 70 nm were synthesized on the surface of the underlying unmodified PbSe film. A peculiar effect was that for the longer treatment durations, when the entire PbSe film was etched away, such isolated PbSe nanoislands were positioned directly on the Si substrate. On the other hand, using PbSe films with granular initial surface, it was possible to fabricate hierarchical mesoporous surficial layers (Figure 1b). Such hierarchical porous networks typically contained large open macropores (100 to 250 nm) with smaller secondary macropores (50 to 75 nm) and mesopores (15 to 20 nm) on the macropore walls. Notably, only a modification of a thin near-surface layer with a thickness of approximately 150 to 200 nm took place during anodic treatment independently of its duration. One important conclusion from this experimental study was that, unfortunately, in both cases, the modified layer occupied only a thin surficial part of the processed PbSe film, and a formation of a well-defined pore network did not take place. It became obvious that the main problem for the results discussed above was the undesirably high etch rate of PbSe that did not allow the formation of a developed thick porous layer due to an active material removal.

**Figure 2:**
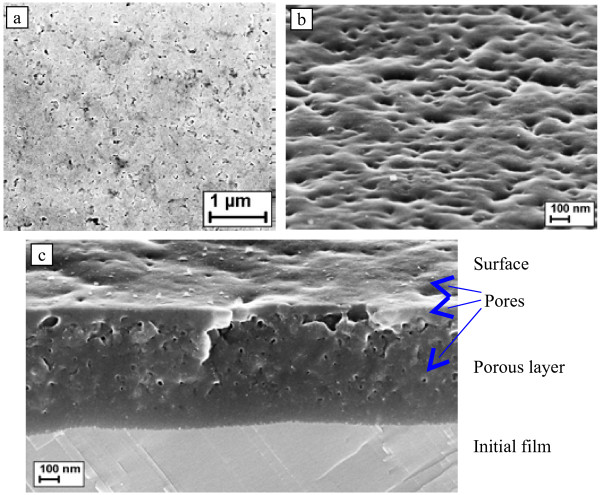
**SEM images of the surface and cross section of the porous PbSe layer.** The surface with normal view ( **a**) and with 70° tilt ( **b**), and the sample cross section with 70° tilt ( **c**).

Therefore, it became necessary to apply a new electrolyte with low-speed electropolishing. We have proposed to introduce to anodic treatment process an electrolyte solution of nitric acid, glacial acetic acid, and glycerol (Tompkins and Johnson solution). Here, nitric acid is the etching agent and acetic acid is the wetting agent that stabilizes the material removal and modification processes. Consequently, the anodic treatment resulted in the formation of well-defined porous layers, showing the success in using glacial acetic acid to limit the dissolution rate. Typical SEM images of a surface and cross section of the PbSe porous layer with a thickness of 750 nm on top of the unmodified PbSe film are given in Figure 2. The average size of the synthesized mesopores was 22 nm, and their density on the surface was approximately 10^10^ cm^−2^. The thickness of the underlying unmodified PbSe film indicated that the thickness of the PbSe layer that was etched away was equal to the thickness of the fabricated porous layer, due to the stabilizing role of glacial acetic acid, in contrast to the Norr electrolyte treatment [8], when the thickness of a porous modified PbSe layer was typically an order of magnitude smaller than the removed layer thickness.

**Figure 3:**
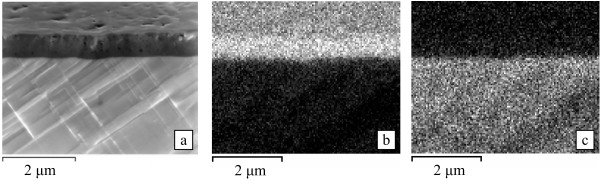
**SEM image of the porous PbSe sample and EDS maps for Se and Pb.** SEM image of the porous PbSe sample cross section (sample tilt is 70°) with the dark porous surficial layer and the underlying initial PbSe film ( **a**); EDS map for Se ( **b**), EDS map for Pb ( **c**).

X-ray diffractometry investigations did not show a formation of any new compounds for the anodized PbSe/CaF_2_/Si(111) samples in addition to the PbSe cubic structure, indicating that the double-layer PbSe structure material is still essentially a monocrystalline PbSe. EDS analysis, however, showed that the stoichiometry of PbSe for the mesoporous layers was severely modified. The EDS chemical mapping based on the L_α1−2_ and M_α1_ rays of the elements lead and selenium showed a high chemical heterogeneity between the porous layer and the underlying unmodified layer (Figure 3). For the initial film, the atomic ratio between Pb and Se atoms on the surface was 0.96, and for the porous layer, it became 0.07, due to the extremely high selenium enrichment. We can assume that during anodic etching, the positive lead ions dissolve into the electrolyte, while the negative selenium ions redeposit on the pore walls. As a result, the matrix remains a monocrystalline PbSe.

**Figure 4:**
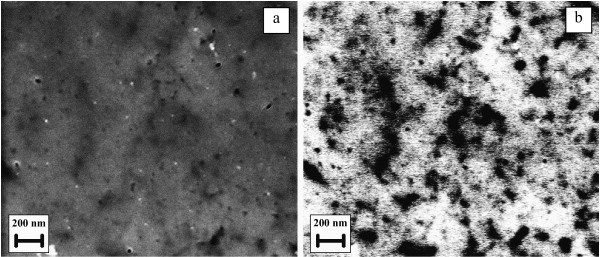
**SEM image and image in back-scattered electrons of the surface of the porous PbSe layer.** SEM image of the surface of the porous PbSe layer ( **a**) and the image in back-scattered electrons with 10-kV high voltage ( **b**) for the same area.

The abovementioned conclusion is supported by the SEM images in back-scattered electrons (Figure 4), which exposed the fact that many of the pores on the surface are obviously closed due to the active redeposition of the products of the electrochemical reactions. More specifically, we observed darker areas around the pores, which became more profound with the increase of the electron energy, meaning that the yield of the back-scattered electrons for these areas decreases. The reason for that is the increase in the proportion of inelastic collisions of electrons with the sample surface. This is possible if the atoms are not rigidly arranged, i.e., have the ability to move under electron impact. Hence, the electrons will pass such a large amount of energy to atoms, while they themselves will lose it and will not be detected as back-scattered. Therefore, this process indicates the ‘soft,’ amorphous regions. We can conclude that for the areas around the pores during anodic etching, atomic Pb leaves the porous layer for electrolyte and the development of an amorphous friable Se layer occurs.

It should be noted that the deposition of chalcogen during electrochemical processings of PbX materials is a known effect, and Se layers were reported to be fabricated electrochemically [11-14]. However, we are the first to observe the effect of selenium redeposition for the electrochemical synthesis of porous PbX layers. Thus, in [12], where Se thin films with PbSe nanoclusters were fabricated electrochemically in nitric acid-based solutions via anodic dissolution of PbSe, the analysis of the corresponding processes showed the reaction of PbSe dissolution to Pb^2+^ ions and Se atoms, which presumably takes place for the porous layers as well. The Se layer on the pore walls can potentially be very beneficial for the practical applications of the porous PbSe layers due to its modified semiconductor properties and high sensitivity in the visible range, and amorphous coatings in particular can have their own specific applications. At the same time, the redeposited Se film can serve as a protective, stabilizing layer by analogy with Te coatings. For example, in [15], PbTe has been coated with a thin sublimed porous elemental tellurium layer, in order to prevent surface oxidation and carbon absorption. Obviously, a further experimental research is required to learn the properties of Se-coated mesoporous PbSe layers fabricated in our study, but there are all reasons to believe that this nanostructured material holds much promise for the fabrication of stabilized porous layer-based devices on silicon wafer substrates.

## Conclusions

In this work, our results demonstrated the potential of the developed novel technique for the synthesis of porous PbSe by anodic electrochemical treatment of PbSe/СaF_2_/Si(111) epitaxial structures in an electrolyte containing glacial acetic acid and nitric acid, with the former wetting the surface and stabilizing the growth of the porous layer. SEM studies showed the presence of mesopores with a surface density of approximately 10^10^ cm^−2^ and an average size of 22 nm, while EDS mapping showed the enrichment of the pore walls with selenium, and the latter effect can be useful for the practical applications of PbSe porous layers in various devices, as well as for the fundamental study of the electrochemical processes during the porous PbX material fabrication.

## Abbreviations

CH3COOH, Acetic acid; EDS, Energy-dispersive X-ray spectroscopy; HNO3, Nitric acid; KOH, Potassium hydroxide; MBE, Molecular beam epitaxy; PbSe, Lead selenide; SEM, Scanning electron microscopy.

## Competing interests

The authors declare that they have no competing interests.

## Authors’ contributions

SPZ designed the research. SPZ, ESG, VVN, and FOS performed the experimental research. SPZ, ESG, and VVN analyzed the data. SPZ and ESG wrote the manuscript. All authors read and approved the final manuscript.

## Authors’ information

SPZ is a professor at the Microelectronics Department, Yaroslavl State University. ESG is a principal engineer at the Microelectronics Department, Yaroslavl State University and a research associate at the Yaroslavl Branch of the Institute of Physics and Technology of Russian Academy of Sciences. VVN is a senior research fellow at the Yaroslavl Branch of the Institute of Physics and Technology of Russian Academy of Sciences. FOS is a student at the Microelectronics Department, Yaroslavl State University.
